# Teachers of various school grades and representations of epilepsy: problems, relational aspects and perspectives of life quality

**DOI:** 10.1186/s13052-015-0177-8

**Published:** 2015-10-05

**Authors:** Giulia Savarese, Luna Carpinelli, Daniela D’Elia, Giangennaro Coppola

**Affiliations:** Department of Medicine and Surgery, University of Salerno, via Allende, 84081 Baronissi, Italy; Centre of Psychological Counseling “Michele Cesaro”, University of Salerno, Baronissi, Italy; Lab H, University of Salerno, Fisciano, Italy; Department of Medicine and Surgery, University of Salerno, Baronissi, Italy

**Keywords:** Concepts, Representations, Teachers, Epilepsy

## Abstract

**Introduction:**

The analysis of the representations of the health of their students by teachers is key to the understanding of the attitudes and behaviors that, in general, take place inside the school community and the educational processes. In fact, social attitudes by teachers and individual within the school environment are often the result of simple categorization and stereotypes, which hinder the process of evolutionary change.

**Aims:**

On these premises it was necessary to investigate the level of knowledge of the epilepsy syndrome, the representations by a group of teachers of the quality of life of people with epilepsy and the representations of the interpersonal relationships between students with epilepsy and their classmates.

**Methodology:**

We used an ad hoc questionnaire, with 33 multiple choice answers, focusing on these variables: work seniority, experience with epileptic students, special education or main teacher.

**Participants:**

The sample consisted of 113 female teachers with a mean age of 44.4.

**Results:**

There aren’t significant differences between the answers of special education and main teachers**:** a) teachers with work seniority > 11 years showed a more than adequate knowledge of information about epilepsy. Moreover, in case of seizure in the classroom, first aid is to call an ambulance rather than administering rescue drugs; b) teachers, who have had a real experience, represent the epileptic student as more limited in the possibilities of getting married, having children, finding a job, and practising a sport; c) teachers, who have had a student with epilepsy don’t report interpersonal relationships with his/her classmates as being difficult. Moreover, they claim to have inadequate knowledge of the educational strategies needed to integrate the epileptic student with his/her classmates; d) there are some different answers for primary school teachers and for secondary school teachers.

**Conclusions:**

Teachers demonstrate inaccurate information about epilepsy, its impact in educational contexts and management of seizures in the classroom. Also, critical areas have emerged indicating efforts should focus on education, sharing the role of teacher, awareness and integration in the class group.

## Introduction

Epilepsy is "*a disorder of the brain characterized by an enduring predisposition to generate epileptic seizures and by neurobiological, cognitive, psychological and social consequences of this condition*" [1, 2]. Such definition highlights the bio-psycho-social aspects related to a disease, which is considered one of the most common in children [3]. The paroxysmal epileptic manifestations, therefore, play an important role in child morbidity for their severity and incidence, which is higher in the early years of the school career [4]. This phase, then, is a critical time for the physical, psychological and social development of the child. For this reason, according to several studies [5–7], the school years have a significant impact on the quality of life of patients with epilepsy and on the social roles they will cover as grownups.

However, some studies [3, 8, 9] have shown that pupils with epilepsy may have a school performance lower than the control sample. Above all, the study by Nuhu, Yusuf Sheikh and Eseigbe [10] showed a positive correlation between poor school performance and three specific variables: early onset (thus longer duration of disease), irregular school attendance and inadequate seizure management. Regarding the earliness of onset of the disease, it is difficult to intervene because of its etiopathological nature. In contrast, absenteeism and the mode of dealing with a seizure within the classroom, call into question the teachers’ knowledge and attitude about epilepsy. For example, with regard to excessive school absences, in the study mentioned above, about 57 % of the pupils with epilepsy reported that they had frequently skipped school because of their disease. Some of these, claiming to choose to stay at home to avoid being mocked by their companions, in case they had a seizure in the classroom. Oftentimes, people with epilepsy are not accepted in class just because others (both classmates and teachers) are afraid to witness the sudden loss of self-control. This can result in a real social rejection [11]. The social stigma associated to epilepsy, which seems to be the most serious and persistent aspect of this disease, is often a serious "handicap" for people living with this condition [12]. This prejudice, influenced by incorrect beliefs and inadequate information about the disease, even on behalf of teachers [13] are quite frequent. Therefore, individuals with epilepsy often face different social problems within the school environment, frequently caused by a negative approach towards them [14, 15].

According to this, the aim of the present study was to examine the information and knowledge about epilepsy, the representations of the quality of life of children with epilepsy and the representations of interpersonal relations between pupils with epilepsy and their classmates, among teachers of various orders and grades, according to their activity and professional experience.

## Materials and methods

A self-report questionnaire, partially modified with respect to Mecarelli et al. [16], consisting of 33 questions with four multiple choice answers was given anonymously to each teacher. It was focused on asking for information concerning the following areas: socio-demographic data (years of employment, whether main or special education teacher, direct experience with students with epilepsy); self-assessment of one’s level of information and knowledge about epilepsy; teacher’ representations of the social implications of the disease.

Statistical analysis was performed by means of SPSS (SPSS, Inc. USA, 2006). Data are expressed as means ± SD. The Student’s *t-*test was used and the p. value less than 0.05 was set as significant.

### Participants

One-hundred and thirteen teachers (all females with an average age of 44.4 years) were participated to the study (primary school, 40; middle school, 40; high school, 33).

Thirty percent were support teachers and 63 % main teachers; thirty-four percent had had direct experience with a student with epilepsy.

## Results

**Fig. 1:**
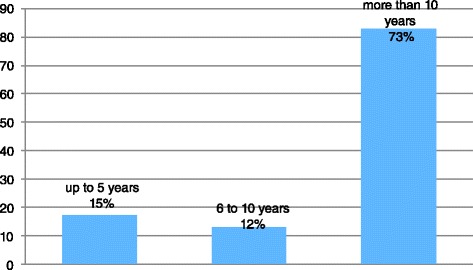
Information on epilepsy held by teachers on the basis of the years of work experience

**Fig. 2:**
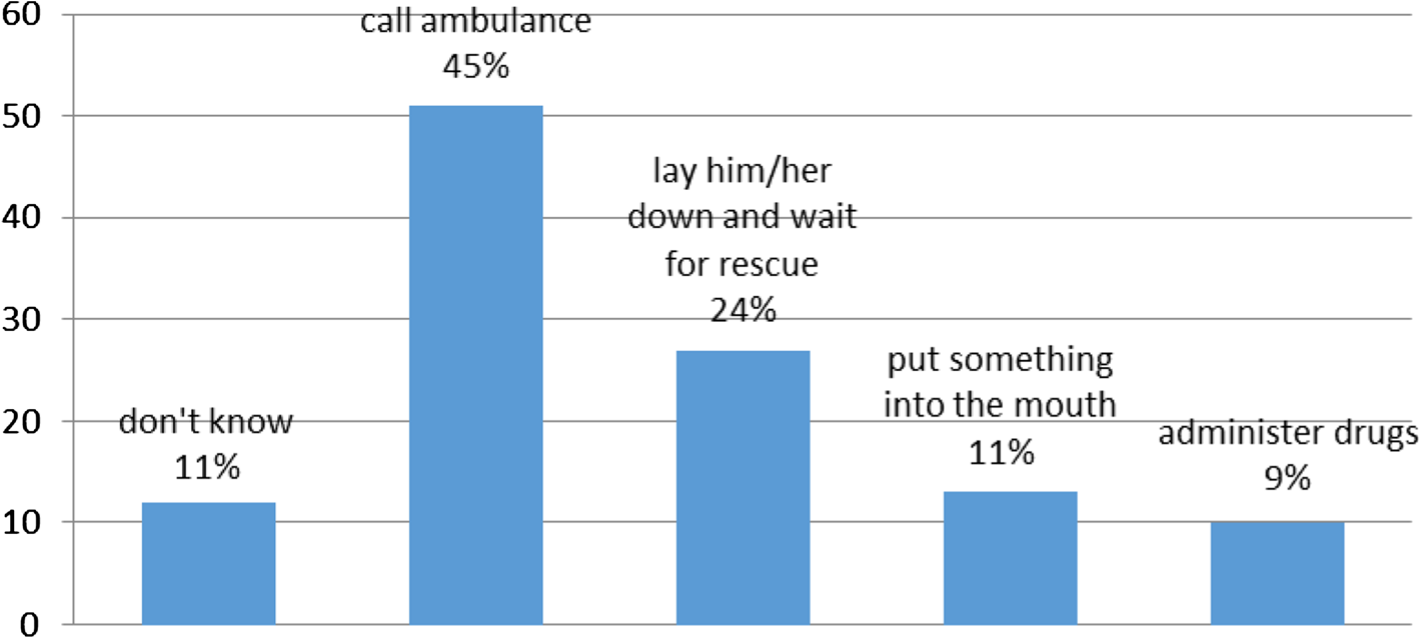
Intervention of the teacher in case of seizure in the classroom

As to the information and knowledge on epilepsy, the teachers having over 10 years of work experience (73 %) demonstrated a more than adequate knowledge on epileptic disorder (Fig. 1). Moreover, in case of seizures in the classroom, 45 % of them offered first aid by calling an ambulance; rescue drugs, were administered by only by 9 % of teachers (Fig. 2).

With respect to the representations of the quality of life of the epileptic person, the teachers, whether or not they had direct experience with a student with epilepsy, showed a representation of their student (Table 1), as an individual being mainly limited in the ability to get married (84 %), procreate (75 %), find a job (55 %), in sports (48 %), and when driving (23 %).

**Table 1:** Obstacles placed by epilepsy

	Yes	No	Don’t know
Marriage	1 %	84 %	15 %
Procreation	5 %	75 %	20 %
Work	34 %	55 %	11 %
Driving	55 %	23 %	22 %
Sports	30 %	48 %	22 %

**Fig. 3:**
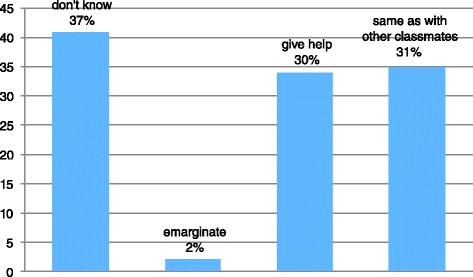
Relationship with classmates

With regards to the representation of the relationship with classmates Fig. 3 shows that the teachers who have had a child with epilepsy in the class tell of interpersonal relationships that are particularly difficult with the group but not marginalizing (61 %).

Finally, the representations of the interpersonal relationships between the epileptic student and his classmates significantly differed (*p* > 0.05) among teachers of various types and grades of schools. Indeed, school teachers were more likely to consider the epileptic children as students like others (60 %) who - through the mediation of all the teachers of the class- constantly interact with their peers and are not marginalized by them. In constrast, teachers in middle and high schools tended to delegate the support teachers (42 %) the task of integrating/involving epileptic pupils, including relational issues with his peers (Table 2).

**Table 2:** Comparison of the responses of teachers of various types and grades (*p* > 0.05 at Student *T* test)

	Primary school	Middle school	High school
The epileptic student is like the other students	60 %	25 %	15 %
The other students do not marginalize	55 %	25 %	20 %
The support teacher has a preferential role for the integration/inclusion of the epileptic pupil	16 %	42 %	42. %

## Discussion

In the present study, a self-report questionnaire anonymously administered to a large cohort of main and support teachers of different orders and grades of school, coming from Campania region, Italy, showed an overall inadequate knowledge of the problems concerning the epileptic disorder as to the relational aspects and perspectives of life quality, as well as an inadequate representation of the epileptic pupil.

In particular, the teachers showed an inadequate knowledge on the administration of the first aid to offer to epileptic pupils. As noted by other studies as well [16], for many teachers "calling an ambulance" in response to a seizure, is a prevalent action, despite the likelihood of this causing potential delays in treatment and subsequent complications for the child [17]. Yet, the current guidelines in several European countries (including Italy) recommend immediate treatment of these children, to prevent the progression of the epileptic status and neurological morbidity [18]. In agreement with these findings, other studies [19, 14] have confirmed that teachers often have little knowledge about the disease inadequate preparation to cope with seizures and misconceptions about epilepsy and its management. Finally, a decisive reason for teachers to refuse to administer rescue drugs for epileptic seizures at school, though often not declared, is the fear of legal implications in the case of "accidents" or "ADRs", for which they simply call the ambulance. Nontheless, the knowledge and skills acquired by each primary school teacher on epilepsy can have a significant impact on the performance and the psychosocial development of children with this disease [20].

In the present study, the teachers interviewed showed a distorted concept regarding the quality of life of children with epilepsy. In keeping with this finding, Bauman et al. [21] reported some teachers considering epilepsy as a factor preventing marriage or causing divorce. Many teachers also questioned the assumption that people with epilepsy can drive a car safely. In contrast, there are a number of studies reporting that subjects with epilepsy can have a happy existence thanks to therapies ensuring seizure control, and are generally satisfied with their lifestyle.

According to the pupils with epilepsy interviewed in this study, classmates are those who would treat them differently, more probably than friends and teachers. Indeed, more than a third of children with epilepsy kept secret in some circumstances their disease to other people, for fear of being treated differently.

Finally, our data support primary school to be the least steeped in prejudice and most integrating in the class context and among peers. Middle and high schools expect that the epileptic pupil is followed by a special education teacher, who should play a preferential role with respect to social inclusion.

## Conclusions

Our data suggests that there are significant gaps within teachers’ general knowledge about epilepsy, its impact in educational settings and the appropriate management of epilepsy and seizures in the classroom. Also, critical areas have emerged where educational efforts within the class group should focus.

It appears necessary to especially encourage specific programs relating to the reception and acceptance of the pupil with epilepsy by the different school components.

In Italy, in this direction, were conducted a series of projects, aimed at raising awareness within schools on the subject 'epilepsy'. In particular, between 2012 and 2013, the Italian League Against Epilepsy (LICE) decided to promote a national campaign to improve knowledge about epilepsy ("Shedding light on epilepsy in school"). The overall objective was to stimulate teachers to cover with pupils the broader issue of diversity, with the intent of making children able to "understand" it without resorting to stereotypes and prejudice. In order to achieve this aim and provide, at the same time, correct information in schools to reduce prejudice about epilepsy, two validated instruments were used for teachers by the LICE: 1) the brochure "Shedding light on epilepsy" containing information about the disorder; 2) posters "If all of a sudden …", on the correct behavior to adopt in the event of a seizure.

More recently (May 2014), the new "Guide to the epilepsies" (including an interactive version), curated by the "Commission promotion" of LICE was freely distributed. It addresses in a comprehensive, yet simple way (thus accessible to adults and children) about twenty topics, from the causes of the disease to the available therapies that allow seizure remission and a better quality of life. The guide also contains practical information (also explained through iconic mediators) on what to do at school if a child has a seizure.

In line with other studies [16], we consider training/informative initiatives like those mentioned above, to be useful in the belief that carrying out educational interventions for the benefit of the population in general and, more specifically, the teachers could increase the diffusion of knowledge on epilepsy, improve the relevant social attitude, promote a higher quality of life for children with epilepsy and their families.
